# Spin and wavelength multiplexed nonlinear metasurface holography

**DOI:** 10.1038/ncomms11930

**Published:** 2016-06-16

**Authors:** Weimin Ye, Franziska Zeuner, Xin Li, Bernhard Reineke, Shan He, Cheng-Wei Qiu, Juan Liu, Yongtian Wang, Shuang Zhang, Thomas Zentgraf

**Affiliations:** 1 School of Physics and Astronomy, University of Birmingham, Birmingham B15 2TT, UK; 2 College of Optoelectronic Science and Engineering, National University of Defense Technology, Changsha 410073, China; 3 Department of Physics, University of Paderborn, Warburger Straße 100, D-33098 Paderborn, Germany; 4 Beijing Engineering Research Center for Mixed Reality and Novel Display Technology, School of Optoelectronics, Beijing Institute of Technology, Beijing 100081, China; 5 School of Computer Science, University of Birmingham, Birmingham B15 2TT, UK; 6 Department of Electrical and Computer Engineering, National University of Singapore, Singapore 117583, Singapore

## Abstract

Metasurfaces, as the ultrathin version of metamaterials, have caught growing attention due to their superior capability in controlling the phase, amplitude and polarization states of light. Among various types of metasurfaces, geometric metasurface that encodes a geometric or Pancharatnam–Berry phase into the orientation angle of the constituent meta-atoms has shown great potential in controlling light in both linear and nonlinear optical regimes. The robust and dispersionless nature of the geometric phase simplifies the wave manipulation tremendously. Benefitting from the continuous phase control, metasurface holography has exhibited advantages over conventional depth controlled holography with discretized phase levels. Here we report on spin and wavelength multiplexed nonlinear metasurface holography, which allows construction of multiple target holographic images carried independently by the fundamental and harmonic generation waves of different spins. The nonlinear holograms provide independent, nondispersive and crosstalk-free post-selective channels for holographic multiplexing and multidimensional optical data storages, anti-counterfeiting, and optical encryption.

## Abstract (web summary)

Metasurfaces offer an approach for computer generated holograms with good efficiency and ease of fabrication. Here, Ye *et al.* report on spin and wavelength multiplexed nonlinear metasurface holography, showing construction of holographic images using fundamental and harmonic generation waves of different spins.

## Graphical abstract

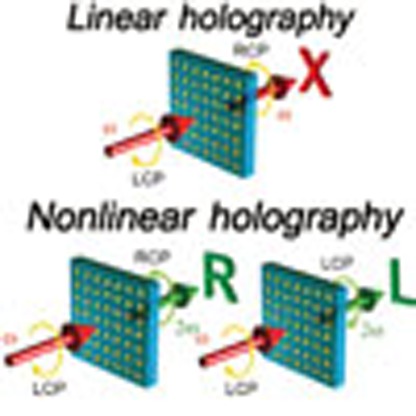

Metasurfaces that consist of an array of function-driven artificial meta-atoms offer an opportunity to create nearly arbitrary designed linear and nonlinear susceptibility distributions^1^,^2^,^3^,^4^,^5^,^6^,^7^,^8^,^9^,^10^,^11^,^12^,^13^,^14^,^15^. The meta-atoms of such tailored metasurfaces are mostly made of metal nanostructures with carefully designed symmetry, orientation and shape. In particular, meta-atoms can be designed to exhibit highly anisotropic responses such that their interaction with electromagnetic fields is strongly polarization sensitive^6^,^7^,^10^. Recently, it was shown that such meta-atoms can store phase information within their orientations, which can be used to alter the propagation of a wavefront^16^,^17^,^18^,^19^,^20^. For the so-called geometric metasurfaces, local geometric phase shifts based on a polarization conversion can be introduced by a simple rotation of the constituent meta-atoms^16^. Benefiting from this convenient local phase control, metasurfaces were successfully applied to demonstrate optical spin–orbit interactions, generating vortex beams, and metalenses^19^,^20^,^21^,^22^,^23^,^24^. Geometric metasurfaces have been recently extended to the nonlinear optical regime for manipulating the phase of the local nonlinear polarizabilities^25^,^26^,^27^. In comparison with the conventional periodic poling process of ferroelectric materials that only gives rise to binary levels of phase, the orientation-dependent nonlinear geometry phase of metasurfaces can be continuous in the entire 2*π* range, providing unprecedented control over the nonlinear generation process.

One important application of metasurfaces is holography. Holography is widely used to record and reconstruct wavefronts by storing and releasing the phase information. To circumvent the challenging phase-recording procedure in conventional holography, computer-generated holograms combine the advantages of computer technology and holography, so that holograms can be designed numerically to generate arbitrary predesignated holographic images of high image quality^28^,^29^,^30^,^31^. Owing to efficient local phase control at the subwavelength scale, metasurfaces offer a novel approach for computer-generated holograms that challenges the conventional depth-controlled holograms in terms of both efficiency and ease of fabrication. More importantly, their subwavelength unit-cell period leads to high resolution with wide diffraction and viewing angles. Recently, three-dimensional, highly efficient, and even broadband holographic imaging has been realized by utilizing metasurfaces^32^,^33^,^34^,^35^. However, an active control of the local phases is indispensable to alter the image or provide dynamic tuning. This might be very difficult to obtain due to the small structure sizes and static properties of the materials being used. Therefore, several techniques for multiplexing information into a single metasurface hologram have been demonstrated, allowing the storage of multiple images for the same illumination wavelength^36^,^37^,^38^,^39^,^40^. The images are either reconstructed at different image planes or encoded in the polarization state of the light, which may result in undesired background light and low image contrast.

Here we demonstrate a nonlinear hologram in which optical information can be multiplexed in both the spin and wavelength spectrum of the output light at the same time. The metasurface hologram is realized by using a Pancharatnam–Berry phase change^41^,^42^, which operates in the linear and nonlinear optical regimes simultaneously^26^. We experimentally demonstrate that the fundamental and second-harmonic optical signals generated from a single-layer metasurface hologram provide an efficient platform to multiplex optical information. Utilizing nonlinear optical processes for holography might lead to techniques in the realization of multicolour image generation or new security identification features.

## Results

### Metasurface design

**Figure 1:**
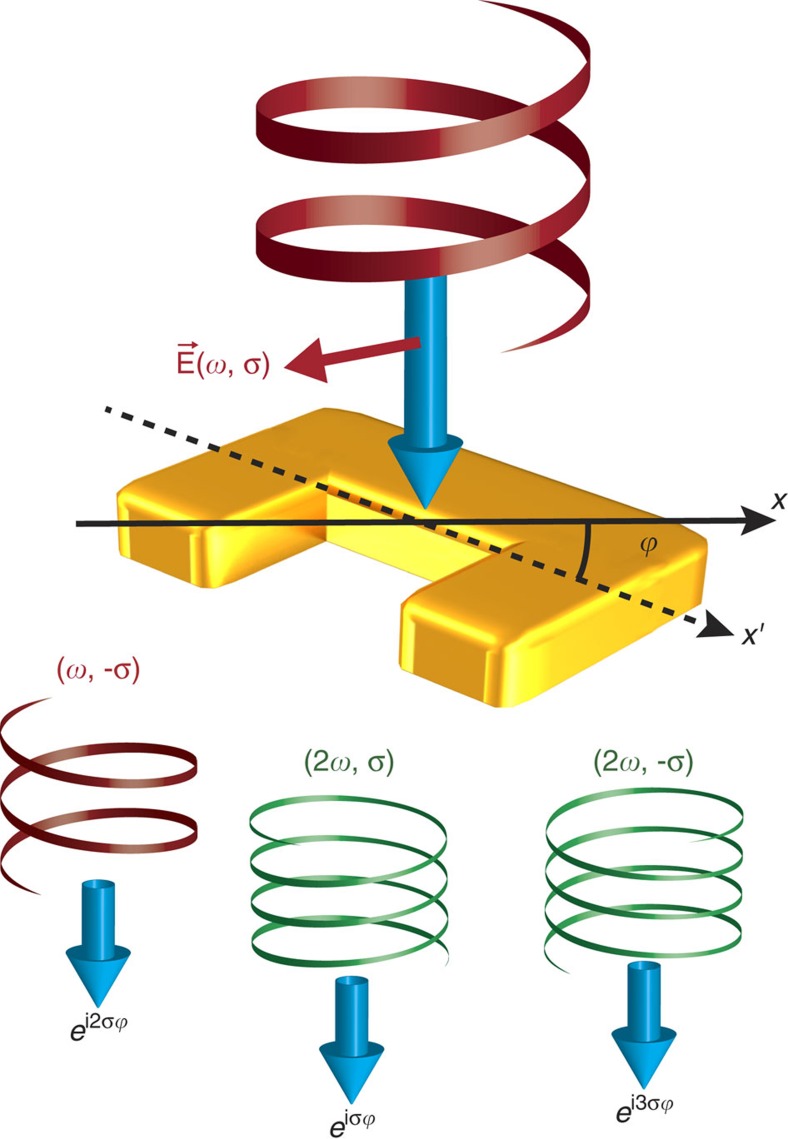
Schematic illustration of the linear and nonlinear geometric phases. For a split ring resonator with an orientation angle of *ϕ*, the co-circular polarized (CP) and cross-CP components of the SHG have nonlinear geometric phases of *σϕ* and 3*σϕ*, respectively, while the transmitted fundamental cross-CP component experiences a linear geometric phase of 2*σϕ.*

To demonstrate the potential of nonlinear phase generation by metasurfaces, we select split ring resonators (SRRs) as meta-atoms due to their strong polarization properties in the linear regime as well as the high second-harmonic generation efficiency^25^,^41^,^42^,^43^,^44^. A circularly polarized incident beam with spin state *σ* is employed as excitation source, where *σ*=±1 stands for left- (LCP) or right circular polarization (RCP), respectively. For a SRR with an orientation angle of *ϕ* (with respect to the laboratory frame), due to its anisotropic response, an optical beam with spin *σ* will experience polarization conversion, resulting in mixed spin states in the transmitted beam. Note that the orientation angle *ϕ*(**r**) is position-dependent. The beam with –*σ* acquires a geometric phase of 2*σϕ* (Fig. 1). The SRR is also a model structure for investigating second harmonic generation due to the lack of centrosymmetry. For a fundamental beam of circular polarization, second harmonic generation (SHG) signals of both spins can be generated due to the lack of rotational symmetry of the SRR. It has been shown previously that the SHG with spin *σ* and –*σ* acquire a nonlinear Berry phase of *σϕ* and 3*σϕ*, respectively (Fig. 1)^45^. Thus, by designing orientation angles *ϕ*(**r**) of each individual antenna in a metasurface, the spatial phase distributions at the metasurface for the transmitted fundamental beam of −*σ*, SHG of spin *σ* and SHG of spin −*σ* are 2*σϕ*(**r**), *σϕ*(**r**) and 3*σϕ*(**r**), respectively. Note that the generated nonlinear phases and the allowed nonlinear conversion processes can be further manipulated by controlling and designing the rotational symmetry^45^,^46^ of the meta-atoms, which could empower many other advanced functionalities.

**Figure 2:**
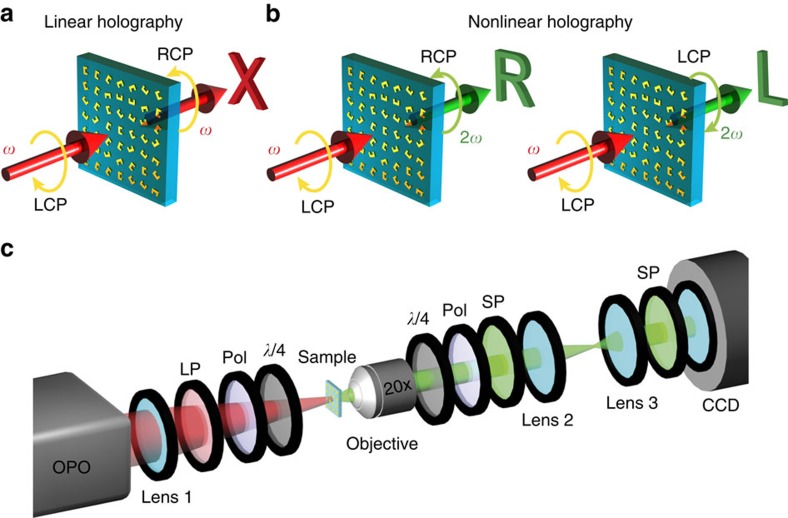
Spin- and wavelength-dependent holographic images. (**a**) Linear optical metasurface hologram for reconstruction of the letter ‘X' at infrared wavelengths. (**b**) Second harmonic generation holographic images for the reconstruction of the letters ‘R' and ‘L' encoded in the different circular polarization states. While the incident wave conditions are kept fixed, the second harmonic LCP wave carries the phase −*ϕ*, while the phase of the RCP wave is −3*ϕ*. (**c**) Schematic illustration of the measurement setup for capturing the holographic images. *λ*/4, quarter waveplate; CCD, camera; LP, long-pass filter; OPO, optical parametric oscillator; Pol, linear polarizer; SP, short-pass filter.

The linear and nonlinear geometric phases, corresponding to the optical signals of different frequencies and different spins, are obviously related since the three phase profiles arise from one single-layer metasurface. Nevertheless, three independent holographic images have been experimentally demonstrated. Such ‘conceptual conflict' however can be explained as follows. First, for most applications only the intensity profile of the holographic image is important and no direct constraint is enforced on the phase profile of the transmitted light. As such, the phase profiles of the three holographic images can be arbitrarily manipulated to optimize the construction of three independent image intensity profiles. The second degree of freedom arises from the fact that two orientation angles of a SRR, which are separated by *π* (*ϕ*,*ϕ*+π), correspond to the same phase profile (2*ϕ*) for the fundamental beam of spin -*σ*, while they lead to different phase profiles for the SHG signals. Similarly, three orientation angles separated by 2*π*/3 (*ϕ*,*ϕ*±2π/3) correspond to the same phase profile (3*ϕ*) for the SHG with spin −*σ*, but distinct phases for SHG with spin *σ*, and fundamental beam with spin −*σ*. Thus, these extra degrees of freedom reduce the correlation between the holographic images formed by the three polarization-encoded linear and SHG signals. In our demonstration, we show that three independent holographic images of the letters ‘X', ‘R' and ‘L' can be constructed at the same spatial area for the three transmitted optical beams with frequency/spin combinations of (*ω*, −*σ*), (2*ω*, −*σ*) and (2*ω*, *σ*), respectively (Fig. 2a,b). We would like to note that the three different images are read out by selecting the frequency and spin of the transmitted light without changing the polarization state of the incident light and the metasurface (Fig. 2c). Here an improved Fidoc algorithm^28^,^29^ is used to design the phase-only nonlinear metasurface CGH that can generate the desired images of reasonably good quality for linear and nonlinear optical signals in **k**-space (for more details see Supplementary Fig. 1, Supplementary Table 1 and Supplementary Note 1).

### Experiment

**Figure 3:**
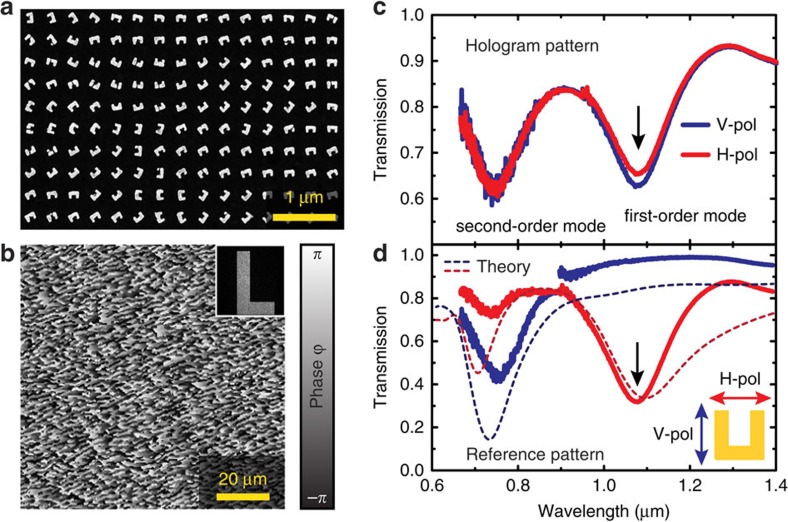
Properties of the fabricated metasurface hologram. (**a**) Scanning electron microscopy image of a part of the fabricated metasurface. Length, width and thickness of the split ring resonators are 160, 130 and 30 nm, respectively. The period between the antennas is 360 nm in both directions. The orientation angles *ϕ* of the structures correspond to the designed phase change along the surface required for the hologram. (**b**) Calculated phase profile for the second-harmonic LCP wave when the fundamental wave is LCP. The inset shows the calculated holographic image from the phase profile on the hologram. (**c**) Measured transmission spectra for horizontal and vertical polarized incident light for the fabricated metasurfaces hologram. (**d**) Transmission spectra for horizontal and vertical polarized incident light of a reference sample with uniformly arranged split ring resonators.

For experimental verification of our nonlinear hologram design, we fabricated SRRs by using electron beam lithography (see Methods section). Figure 3a shows the scanning electron microscopy image of the fabricated metasurface with the arrangement and orientation of the SRRs for a small area across the metasurface. The distribution of the orientation angle among the SRRs results in a phase distribution for the linear and nonlinear signals as discussed above. An example of the corresponding phase profile of the SHG signal with the same spin (LCP) as that of incident beam is shown in Fig. 3b. Since the efficiency for SHG strongly depends on the resonant excitation of the localized Plasmon modes within the antennas, we measured the polarization-dependent linear transmission spectra of the fabricated metasurface hologram for both horizontal and vertical polarizations to determine the resonance wavelengths (Fig. 3c). The SRRs are designed to have a fundamental plasmonic mode around a wavelength of 1,100 nm (ref. 47). The measurement shows the first-order mode at 1,080 nm, which only slightly blue-shifted relative to the simulation. Due to the nearly random distribution of the orientation angles of the SRRs across the metasurface, both polarization measurements give nearly identical transmission spectra. The strongest SHG intensities are expected for fundamental wavelengths around the resonance wavelength of 1,080 nm. Furthermore, there is a second-order resonance for vertical polarization around 745 nm. As the resonance of this higher mode is considerably far away from the range where the SHG signals will be generated, we do not expect any impact of this resonance on the SHG efficiency from the metasurface. For practical purposes the SHG conversion efficiency of the SRRs can be further increased by incorporating highly nonlinear materials into the metasurface design. Recently it was shown that coupling the plasmon resonances to intersubband transitions of multiple quantum wells can enhance the SHG signal several orders of magnitude^48^. In contrast, a reference sample containing only aligned SRRs shows a more pronounced polarization dependence. Here the first-order resonances of the SRR appears only for horizontally polarized incident light, while the second-order resonance shows up for vertical polarizations^47^.

**Figure 4:**
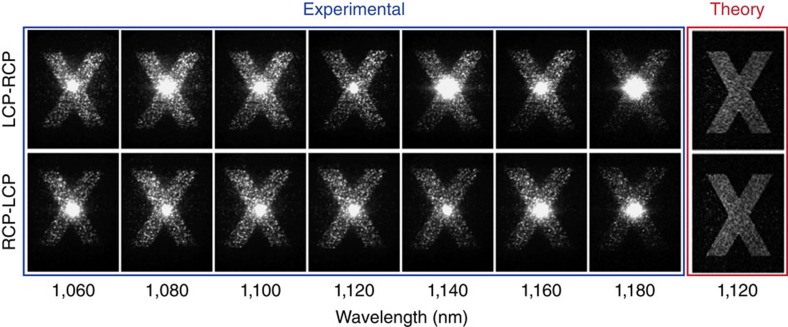
Experimental investigation of holography at the fundamental wavelengths. Linear holographic image at various fundamental wavelengths is recorded when incident and measured polarizations are perpendicular. The simulated reconstructed images from the perfect phase hologram at 1,120 nm are shown at the right.

**Figure 5:**
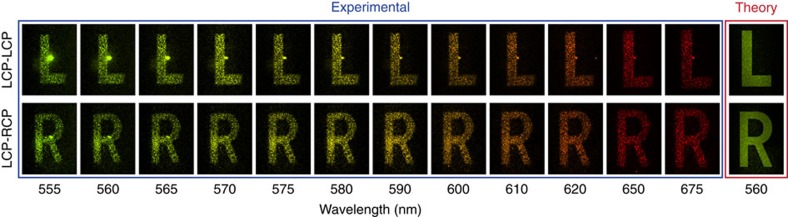
Experimental investigation of nonlinear holography at the second harmonic frequency. Two-channel holographic images at second-harmonic wavelengths. Note that the camera images are post-processed with false colour based on the wavelength for better illustration of the results (for more details see Supplementary Note 2). Theoretically expected results for the SHG holographic image at 560 nm are shown at the rightmost column.

First we reconstruct the holographic image of the hologram for the fundamental wavelength over a broad wavelength range (Fig. 4). The holographic image produced in the Fourier space has an opposite spin to that of the incident beam. Here we measured the cross-polarized holographic image for both incident circular polarization states. Note that for illuminations with opposite circular polarizations, the holographic images are conjugate to each other, that is, they are symmetric about the origin in the Fourier space. Since the image itself is centrosymmetric, there appears to be no difference between two conjugate images. With the increase of the wavelength, the image size slightly increases due to the constant geometric phase for all wavelengths and the fixed period of the antennas. The bright spot in the centre of the image results from the undesired scattered light of the metasurfaces due to imperfections of the antenna structures as well as some remaining transmitted light at the incident polarization. The highest conversion efficiency for the incident light at the metasurfaces is obtained for wavelength around 1,100 nm. The efficiency for the metasurfaces hologram is significantly reduced for wavelength far away from the resonance wavelength of the antennas, and consequently the ratio between image intensity and undesired background signal is also reduced. The most right panel in Fig. 5 shows the target image based on simulation for comparison.

We next measure the spin-dependent nonlinear holographic images of the metasurface hologram at the SHG wavelengths (Fig. 5). By using a quarter-waveplate and a linear polarizer in front of the detector, SHG holographic image of each spin can be selectively obtained. For LCP light illumination at the fundamental wavelength the LCP-SHG signal of the metasurface generates an image with the letter ‘L' in **k**-space, whereas the RCP-SHG signal generates a letter ‘R'. Hence two different images are reconstructed by the nonlinear signals of opposite spins. The bright spot in the centre of the LCP–LCP images results from an insufficient suppression of the fundamental wave, which is also left circularly polarized. For the SHG signal of RCP polarization, an additional cross-polarization filter removes the fundamental background. For illumination wavelengths away from the resonant excitation of the antennas, the SHG image intensity drops and results in a reduced signal-to-noise ratio at the detector. Nevertheless, the wavelength range for obtaining a sufficient signal-to-noise ratio is much larger than for the fundamental wavelengths since no background signal at the SHG exists and the fundamental wavelength can be filtered out more efficiently. Hence the nonlinear generated images at the SHG wavelength in the visible range are very well suited for traditional silicon CCD detectors, as these detectors have a low sensitivity in the NIR but are very sensitive in the VIS.

**Figure 6:**
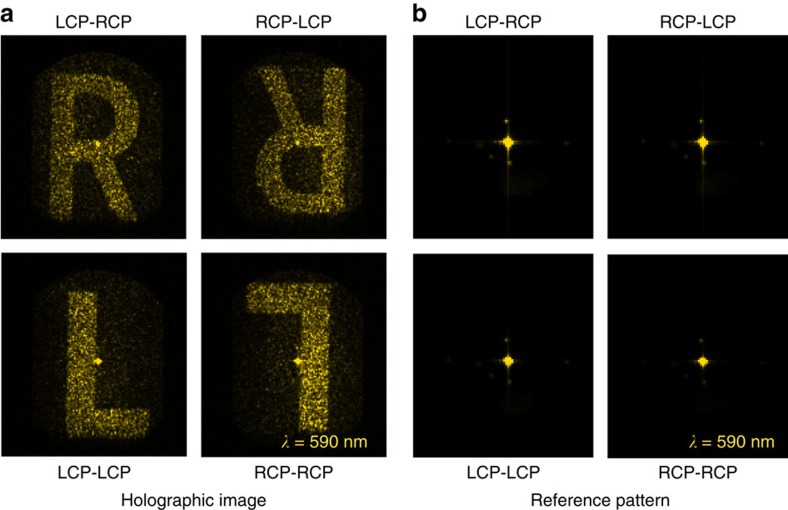
SHG holographic images for different combinations of the spins of the incident and output beams. (**a**) Recorded Fourier plane SHG holographic images at 590 nm of the hologram pattern for various combinations of the incident and SHG circular polarization states. For a simultaneous change of the incident and output polarizations the sign of the geometrical phase changes, resulting in a conjugate image. (**b**) Recorded Fourier plane SHG holographic images for the reference metasurface show dominantly SHG signal at the centre of the **k**-space.

Finally, we measure the holographic images for all the combinations of the incident circular polarization and the SHG polarization for the SHG wavelength at 590 nm (Fig. 6a). By simultaneously flipping the spins of both the incident fundamental beam and the detected SHG beam, we obtain the conjugated images as the sign of the phase is reversed. Hence the two conjugate images appear to be symmetric to each other about the origin of the **k**-space. For comparison the same measurement is performed on the reference metasurface where all SRRs have the same orientation and generate therefore a homogeneous nonlinear phase (Fig. 6b). As expected the **k**-space images show only a strong SHG intensity at the centre of the **k**-space.

## Discussion

We demonstrated that by utilizing nonlinear optical processes (second harmonic generation) multi-channel information capacity can be obtained for a single metasurface. Here the design of the nonlinear hologram benefits from the unique linear and nonlinear optical properties of geometric phase metasurfaces. The various information channels can be used to encode different images within a single hologram, whereas the observation conditions determine which information channel is read out and not by the illumination conditions. Thus, with the nonlinear frequency conversion more information channels can be introduced to provide more independent information than in linear optical holography^32^,^33^,^34^,^35^,^36^,^37^,^38^,^39^,^40^. Though our CGH is designed to reconstruct images in the far field (**k**-space), the propagation function in the algorithm suggests that images can be also reconstructed in the Fresnel zone of the hologram, allowing multiplexed nonlinear three-dimensional image generation. Nonlinear metasurface holography is a promising candidate for applications in anti-counterfeiting, hidden security identification features and optical encryption. With future development in the enhancement of the conversion efficiency by utilizing highly nonlinear material systems, nonlinear holography might be even suitable for data storage and information display technology.

## Methods

### Sample fabrication and experimental setup

The holograms were fabricated on an ITO-coated glass substrate with standard electron-beam lithography, subsequent deposition of 2 nm chromium as adhesion layer and 30 nm gold, and lift-off process. Both linear and nonlinear imaging was done by focusing a circularly polarized femtosecond-laser beam generated by an optical parametric oscillator of desired wavelength with an achromatic lens (*f*_1_=300 mm) to a spot size diameter of ∼150 μm. The lasers repetition rate was 80 MHz, its pulse duration ∼200 fs and the used average power 100 mW for the linear imaging and 350 mW for the nonlinear imaging. For the nonlinear imaging this corresponds to an average pump intensity of ∼2.3 kW cm^−2^. The metasurface was illuminated at normal incidence and the generated linear and nonlinear signals were collected by an infinity corrected microscope objective (× 20 magnification and NA=0.45). Filters are used to discriminate the pump laser while extracting one of the nonlinear holograms with a polarization analyser. Two lenses (*f*_2_=100 mm and *f*_3_=35 mm) were used to image the Fourier plane after the microscope objective onto a thermo-electrically cooled CCD detector (Andor iDus). We captured the near-infrared holographic image by substituting the visible by a near-infrared polarization analyser and removing the short-pass filters.

### Data availability

The data that support the findings of this study are available from the corresponding authors upon request.

## Additional information

**How to cite this article:** Ye, W. *et al.* Spin and wavelength multiplexed nonlinear metasurface holography. *Nat. Commun.* 7:11930 doi: 10.1038/ncomms11930 (2016).

## Supplementary Material

Supplementary Information Supplementary Figure 1, Supplementary Table 1, Supplementary Notes 1 & 2 and Supplementary References.
