# Supplementary material for: Spin and wavelength multiplexed nonlinear metasurface holography
Source: Nat Commun. 2016 Jun 16;7:11930. doi: 10.1038/ncomms11930 (PMC4912630; doi:10.1038/ncomms11930)
Supplement: Supplementary Information — Supplementary Figure 1, Supplementary Table 1, Supplementary Notes 1 & 2 and Supplementary References. [file ncomms11930-s1.pdf]

Supplementary Figure 1

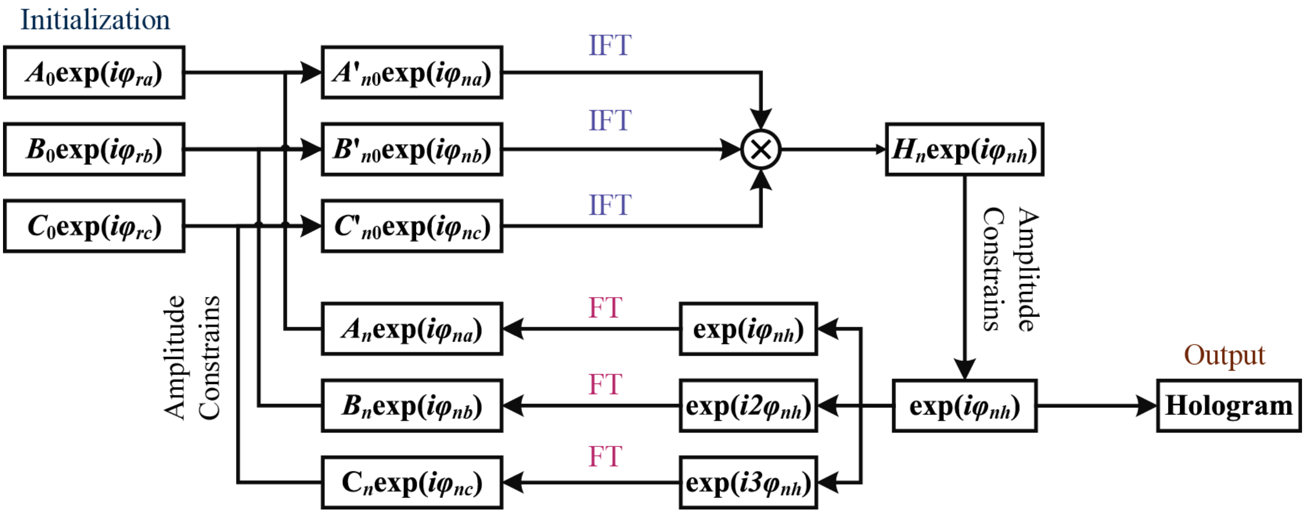

Flow chart for the hologram generation with a circularly polarized incident beam of frequency ω and helicity σ. The hologram generation start with an initialization by adding random phases φ_r to every target amplitude distribution A_0, B_0 and C_0, where A_0, B_0 and C_0, represent amplitude distributions of the target images for frequency-helicity combination of (2ω, σ), (ω, -σ) and (2ω, -σ), respectively. After an inverse Fourier transform, the complex amplitude distributions for φ, 2φ, and 3φ on the holographic image plane are combined together. To achieve phase-only holograms, an amplitude constraint is introduced, where the amplitude of the combined distribution is set to unity. After several iterations, the final target phase-only distribution is acquired. A_n, B_n, C_n, A'_{n0}, B'_{n0} and C'_{n0}, refer to the amplitude distribution before and after applying the amplitude constraints in the nth iteration. FT and IFT stand for Fourier transformation and inverse Fourier transformation, H_n and φ_{nh} describe the amplitude and phase after the combination of three holograms.

Supplementary Table 1

Image	PSNR/dB
"L" (φ)	32.4880
"X" (2φ)	34.9013
"R" (3φ)	32.1518

Calculated peak signal to noise ratio for the evaluation of the image quality. Here we employ a widely used metric – peak signal-to-noise ratio (PSNR) for the evaluation of the holographic image quality. The PSNR is defined as the ratio between the maximum possible intensity of the image under evaluation and the corrupt influence by noise, which is quantified by the mean squared error. The PSNR of the three simulated images carried by different wavelengths and polarizations are shown in Supplementary Table 1. Note that the common acceptable value of the PSNR is 30 dB.

Supplementary Note 1: Design of the Computer Generated Hologram

We use the split ring resonator (SRR) - a C1 rotational symmetric structure, as the basic building block of the hologram, since both second harmonic signals with same and opposite circular polarization states compared to the fundamental beam are allowed. We like to note that in a bulk material with C1 rotational symmetry around the light propagation direction, circular polarized light experiences a change of the polarization state during its propagation. Therefore, the selection rule for C1 symmetry may lose its meaning for thick materials. On the other hand the metasurface is very thin and propagation effects do not play any role. Hence, the selection rule for C1 can be applied, with the allowed orders of harmonic generation given by $m = nl \pm 1$, with l being an arbitrary integer, n representing the symmetry order, and the '+' and '-' sign corresponding respectively to harmonic generation of the same or opposite circular polarization state as the that of the fundamental beam. The above equation shows that for a C1 structure (n = 1), any orders of harmonic generation are allowed for both circular polarizations.

We employ a modified Fidoc algorithm to obtain the desired geometric phase of the metasurface[1]. The Fidoc algorithm is an optimized iteration phase retrieval algorithm, which is widely used to obtain the phase-only Computer Generated Hologram (CGH) in space domain and reconstruct the desired images as the target distributions of the transmitted lights in k-space (Supplementary Figure 1)[2,3]. Starting with a random phase distribution $\varphi_0(x,y)$, three channels with spatial distributions $e^{i\varphi_0(x,y)}$, $e^{i2\varphi_0(x,y)}$, and $e^{i3\varphi_0(x,y)}$ are Fourier transformed into k-space. In the next step the three constraints on the amplitude distributions, corresponding to three desired holographic images, are multiplied to the three channels. Then, they are transformed back into the space-domain by the inverse Fourier transformation. In the space domain, we introduce the dependent relationship (φ, 2φ, and 3φ) among the phase distributions to the three channels. The new phase distribution $\varphi_1(x,y)$ is used for the next iteration. After several iterations, the phase profiles converge and we can finally acquire the desired phase-only distribution as the hologram. This phase distribution is then translated to the orientation angle of a periodic pattern of SRRs on the surface.

Considering the correlation between the three Pancharatnam-Berry phases in the linear and nonlinear signals, one certainly cannot independently optimize the holographic image carried by one channel without affecting the images carried by the other two channels. On the other hand, the iteration algorithm maximizes the quality of the images by exploiting the following fact: the quality of the images is only determined by their intensity profile, whereas the phase profiles of the holographic images can be arbitrary, and therefore can serve as free parameters for optimization of the image quality, and consequently minimizing the cross-talk between the three holographic images.

Supplementary Note 2: False color rendering in figures

The intensities of holographic images are recorded in experiment by a gray scale Si-CCD camera, and false colors are introduced in Figs. 5 and 6 based on the utilized wavelength for better visualization for the human eyes. We used the values for the red (R), green (G), and blue (B) channels that are given by Dan Bruton's approximations to R, G and B as a function of wavelength[4]. Then the images are rendered in image processing software to adjust the maximum intensity according to the RGB values. Though the RGB definition are different from that in W. D. Wright or J. Guild experiments and that in CIE system[5], one can still

approximately evaluate the results as expected.

Supplementary References

1. Georgiou, A., Christmas, J., Collings, N., Moore, J. & Crossland, W. A. Aspects of hologram calculation for video frames. *J. Opt. A-Pure Appl. Op.* **10**, 035302 (2008).
2. Akahori, H. Spectrum leveling by an iterative algorithm with a dummy area for synthesizing the kinoform. *Appl. Opt*. **25**, 802-811 (1986).
3. Jia, J. et al. Reducing the memory usage for effective computer-generated hologram calculation using compressed look-up table in full-color holographic display. *Appl. Opt.* **52**, 1404-1412 (2013).
4. efg's Computer Lab, Spectra Lab Report. http://www.efg2.com/Lab/ScienceAndEngineering/Spectra.htm (2006).
5. Schanda J., *Colorimetry: Understanding the CIE System* (John Wiley & Sons, Inc., 2007) pp. 25-78.
